# Supplementary material for: High-lying valley-polarized trions in 2D semiconductors
Source: Nat Commun. 2022 Nov 15;13:6980. doi: 10.1038/s41467-022-33939-w (PMC9666447; doi:10.1038/s41467-022-33939-w)
Supplement: Supplementary file 1 — Supplementary information [file 41467_2022_33939_MOESM1_ESM.pdf]

Supplementary Materials for

High-lying valley-polarized trions in 2D semiconductors

Kai-Qiang Lin[1]*, Jonas D. Ziegler[2], Marina A. Semina[3], Javid V. Mamedov[4], Kenji Watanabe[5], Takashi Taniguchi[6], Sebastian Bange[1], Alexey Chernikov[2], Mikhail M. Glazov[3,4]*, John M. Lupton[1]

*Correspondence to: kaiqiang.lin@ur.de (K.-Q.L.), glazov@coherent.ioffe.ru (M.M.G.)

Contents:

Supplementary Note 1: Experimental results
Supplementary Note 2: Rationalization of the helicity of the high-lying excitons and trions
Supplementary Note 3: Theoretical considerations of trions with negative-mass electrons
Supplementary Figs. 1-12
Supplementary Table 1
Supplementary References (1 to 23)

CONTENTS

Note 1. Experimental results 2

Note 2. Rationalization of the helicity of the high-lying exciton and trions 8

Note 3. Theoretical considerations of trions with negative-mass electrons 13

A. Model of parabolic dispersion 13

B. Effects of band non-parabolicity 15

C. Scattering theory approach and high-lying Fermi polarons 18

References 19

Supplementary Note 1: Experimental results

Transition-metal dichalcogenide monolayer transistors can suffer from strong hysteresis when using silicon as the back gate and a silicon dioxide layer as the insulator. This hysteresis is due to charge trapping in the silicon dioxide layer and can be prevented by using hexagonal boron nitride (hBN) layers as the insulator and few-layer graphene as the gate instead. Supplementary Fig. 1a,b presents the gate dependence of photoluminescence (PL) from monolayer WSe_2 for different voltage sweeping directions, showing no hysteresis. Supplementary Fig. 1c,d shows the reflectance derivative of the monolayer WSe_2 sample as a function of the gate voltage. The 1*s* (Supplementary Fig. 1d) and 2*s* (Supplementary Fig. 1e) states of the band-edge A exciton blueshift with increasing the charge density. Simultaneously, charged 1*s* and 2*s* states emerge[1,2].

To support the argument that Auger-like exciton-exciton annihilation process is responsible for the HX UPL, we perform a pump power dependence measurement of both the UPL and the continuous-wave second harmonic generation (SHG) in Supplementary Fig. 2a. For the Auger-like exciton-exciton annihilation process, the UPL of the HX should scale superlinearly with the exciton population density. However, because of a bleaching of the absorption band, the A-exciton density scales sublinearly with the pump power at the high pump fluences. Therefore, the UPL intensity of the HX transition is not necessarily quadratic in the pump power. Nevertheless, second-harmonic generation (SHG) generally scales parabolically with the excitation power and therefore serves as a suitable reference in the measurement when it is resonantly enhanced by the A-exciton transition. We plot both the continuous-wave SHG and the HX UPL intensity a function

of the pump power in Supplementary Fig. 2b. The UPL intensity of the HX shows a power-law exponent of 1.26±0.04, which is very close to that of 1.35±0.04 found for the SHG intensity measured simultaneously (reduced from the canonical exponent of 2 because of the bleaching of the A-exciton transition), thus supporting the notion of an Auger-like exciton-exciton annihilation process being responsible for the HX UPL.

To enhance the contrast of the charged high-lying exciton (HX) features in gate-voltage dependent upconverted PL (UPL), we plot the UPL as a function of gate voltage after subtracting the UPL spectrum at the charge neutrality point (-0.2 V) in Supplementary Fig. 3.

As shown in Fig. 2 and Supplementary Fig. 1d, the UPL of high-lying trions vanishes at high charge densities. This disappearance occurs because the A exciton energy blueshifts as the charge density increases as illustrated in Supplementary Fig. 1d and we use a constant excitation energy for the gate-voltage dependent UPL measurement. Supplementary Fig. 4 plots the helicity of the two-photon luminescence (TPL) of high-lying trions from monolayer WSe_2, showing that the valley polarization of high-lying trions does not decrease with an increase of charge density.

Supplementary Table 1 summarize the binding energies of the neutral and charged band-edge A excitons as well as the binding energies of neutral and charged high-lying excitons.

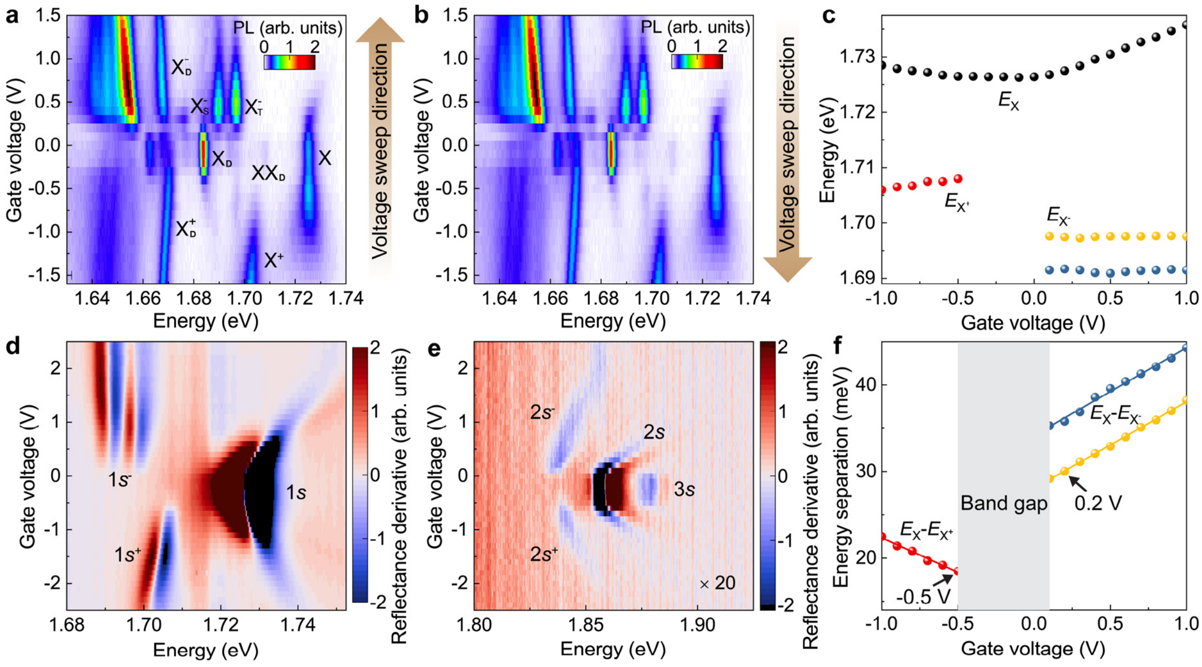

Supplementary Fig. 1 | PL and reflectance spectra as a function of gate voltage for a monolayer WSe_2 transistor device. Dependence of the PL spectrum on gate voltage, (**a**) when sweeping from -1.6 V to 1.5 V and **(b)** when sweeping from 1.5 V to -1.6 V. No hysteresis is observed. (**c**) Peak energies of the neutral and charged excitons. (**d, e**) Derivative of the reflectance contrast of the monolayer WSe_2 device at different gate voltages, showing the neutral and charged 1*s* state of the A exciton (d) and the excited A:2*s* exciton (e). (**f**) Energy separation between neutral and charged excitons in panel c as a function of the gate voltage. Linear fits (lines) mark the change of the energy separation due to the change of the Fermi level[1,3]. At 0.2 V and -0.5 V, the energy separations and thus the Fermi levels are close to their values at the band edge.

Supplementary Table 1. Summary of binding energies of neutral and charged high-lying (HX) and band-edge (X) exciton species for monolayer WSe_2.

Excitons	X	X	X^+	X_S^-	X_T^-
Binding energy (meV)	450 (theory)[4] (w/o hBN)	~167 (experiment)[5]	21 (experiment)	36 (experiment)	29 (experiment)
Excitons	HX		HX^+	HX^-	
Binding energy (meV)	600 (theory) [4] (w/o hBN)		35 (experiment)	43 (experiment)	

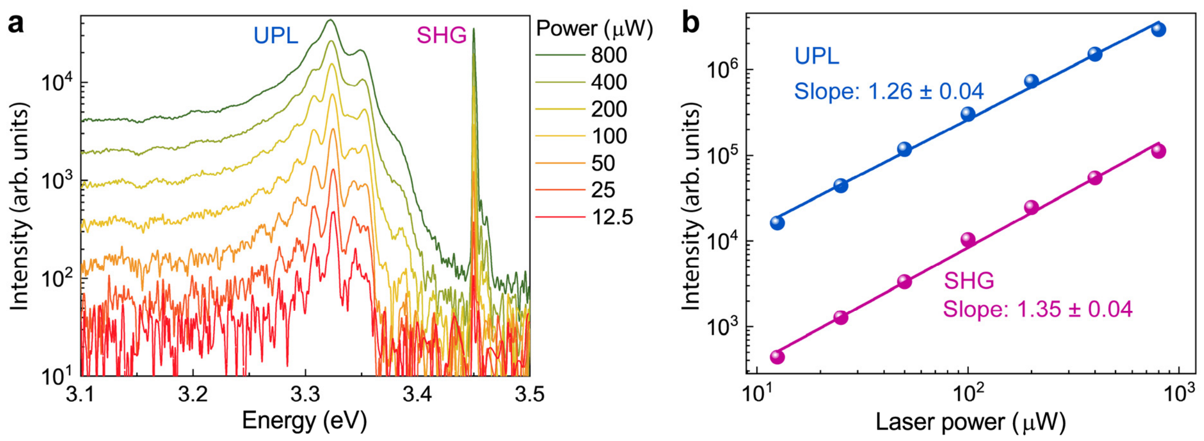

Supplementary Fig. 2 | Pump power dependence of upconverted photoluminescence (UPL) and continuous-wave second-harmonic generation (SHG). a, Simultaneous measurement of the UPL and SHG from monolayer WSe_2 encapsulated by hBN at 5 K as a function of excitation power of the narrow-band continuous-wave laser at 1.726 eV. **b,** Power dependence of the integrated HX UPL and SHG intensity. The HX UPL shows a power law almost identical to that of the SHG, suggesting an Auger-like exciton-exciton annihilation process for the UPL, and both UPL and SHG are impacted by the bleaching of the A-exciton absorption at high fluences.

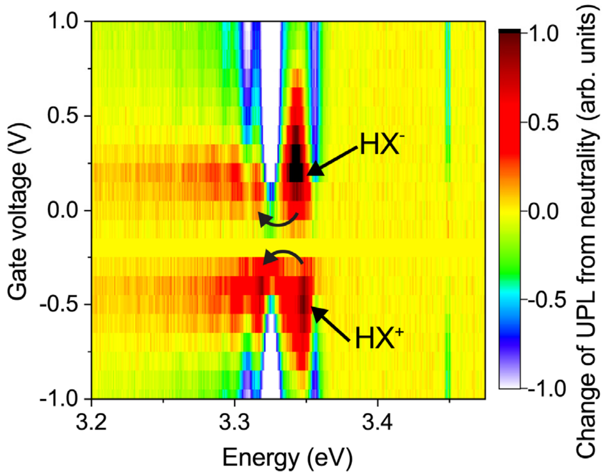

Supplementary Fig. 3 | Change of UPL intensity from the charge neutrality point. Black arrows mark the charged high-lying excitons and their corresponding phonon progression.

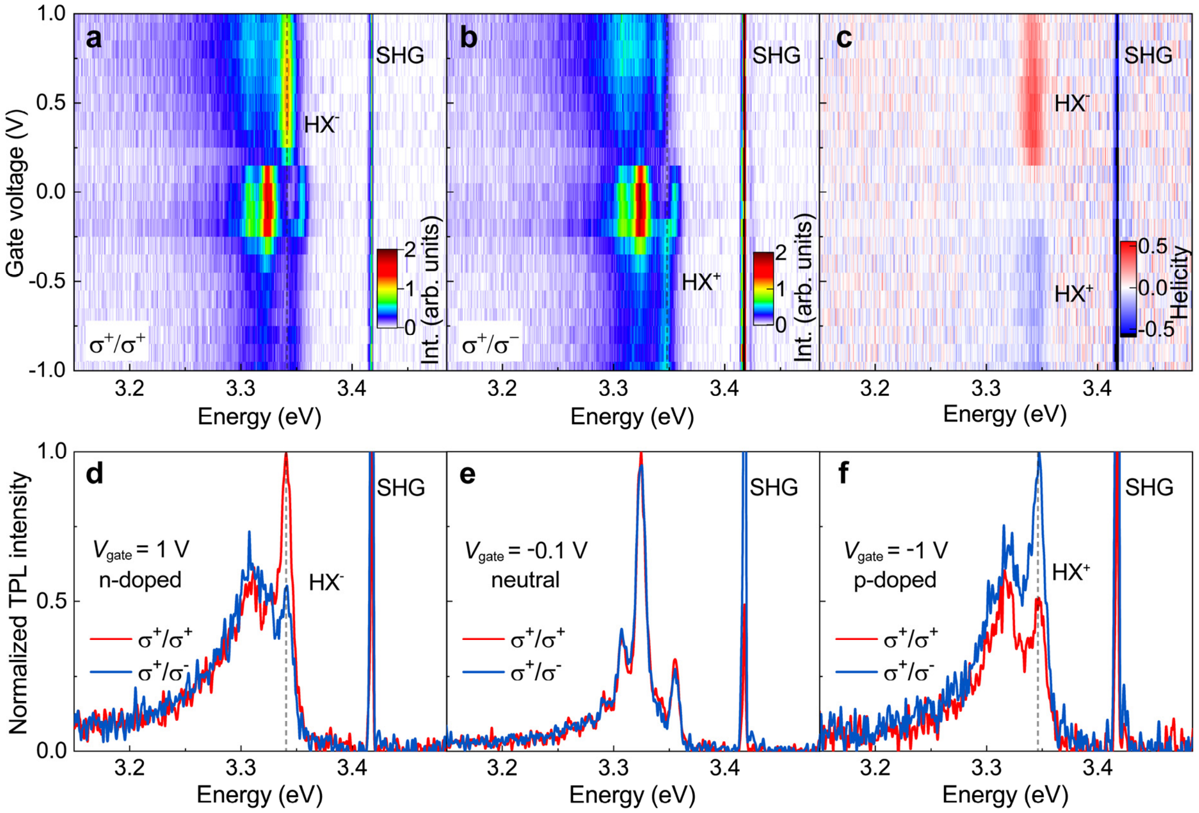

Supplementary Fig. 4 | Helicity of the two-photon luminescence (TPL) of high-lying trions from monolayer WSe_2. (**a, b**) Gate-voltage dependence of co-polarized (**a**) and cross-polarized (**b**) two-photon PL (TPL) under left-hand circularly polarized, continuous-wave excitation at 726 nm. (**c**) Helicity of the TPL as a function of gate voltage. (**d-f**) Polarization-resolved TPL spectra at gate voltages of 1 V (**d**), -0.1 V (**e**), and -1 V (**f**).

Supplementary Note 2: Rationalization of the helicity of the high-lying exciton and trions

In this section, we explain the helicities of the high-lying exciton and trions in monolayer WSe_2 based on the assumption that annihilation processes between band-edge intervalley excitons or intervalley excitons and trions constitute the dominant mechanism for forming high-lying excitons and trions. This assumption is supported by the evidence in Ref. 6 that intervalley excitons are the dominant species responsible for annihilation processes in monolayer WSe_2 at low temperatures.

Supplementary Fig. 5 illustrates the mechanism of the annihilation between these intervalley excitons, leading to a high-energy exciton at the ±K valleys rather than a K-Γ exciton. The two annihilation processes in Supplementary Fig. 4b are symmetric, with equal probability, and therefore lead to an equal amount of the neutral high-lying excitons at the +K and -K valleys.

This symmetry breaks down in the annihilation process between intervalley excitons and trions. As shown in Supplementary Fig. 6 for the case of electron doping, σ^+ photons excite excitons at the +K valley and polarize resident carriers with spin down at the -K valley[7]. This resident carrier polarization effect leads to more intervalley trions forming with resident electrons at the -K valley rather than at the +K valley. The annihilation involving such trions, illustrated in the lower panel of Supplementary Fig. 5b, gives rise to negative high-lying trions at the -K valley with σ^+ polarization.

Supplementary Fig. 7 shows the hole doping situation, where σ^+ photons pumping in the +K valley create intervalley trions with resident holes at the -K valley. The annihilation process between such intervalley trions and an intervalley exciton with opposite momentum gives rise to positive high-lying trions at the +K valley with σ^- polarization.

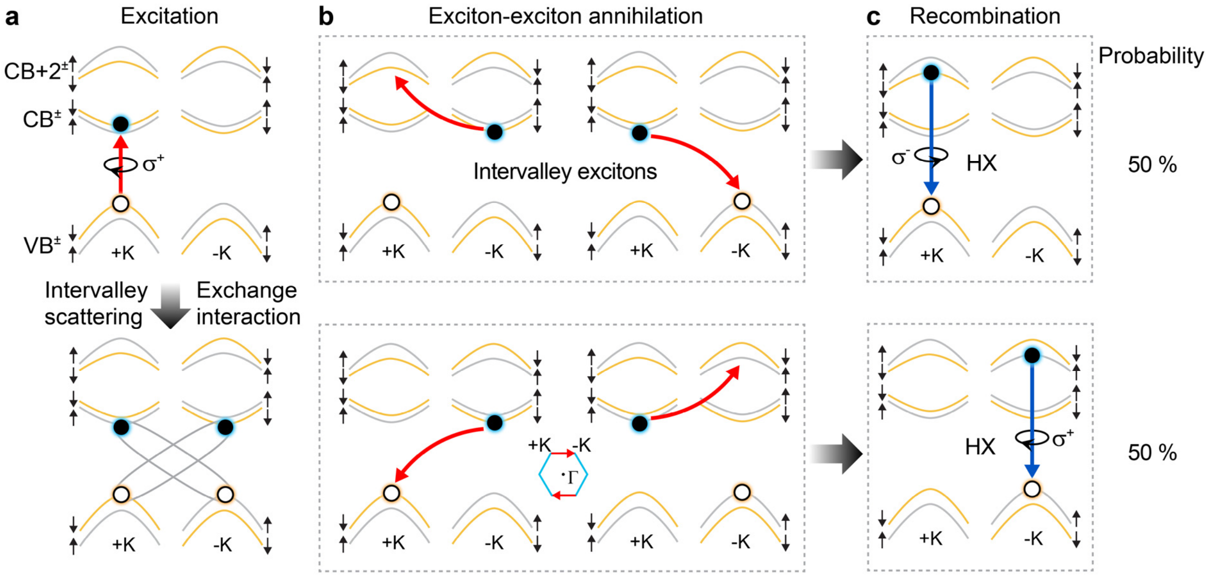

Supplementary Fig. 5 | Annihilation between intervalley excitons for the formation of neutral high-lying excitons. a, A σ^+ photon selectively excites the exciton at the +K valley and, after a fast exchange interaction and intervalley scattering processes, intervalley excitons form. **b,** Exciton-exciton annihilation occurs between intervalley excitons with opposite momentum, conserving energy and momentum. The processes in the upper and lower panels are symmetric and occur with equal probability. **c,** High-lying excitons form in the +K and -K valleys with equal probability and show no helicity.

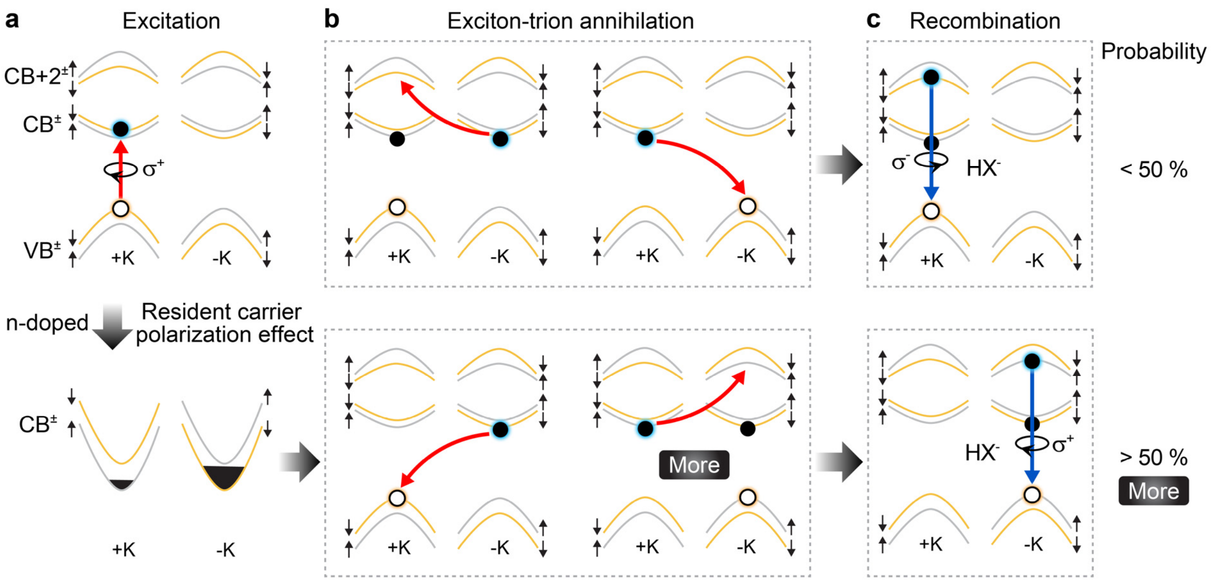

Supplementary Fig. 6 | Annihilation between an intervalley exciton and a trion for the formation of negative high-lying trions. a, Resonant excitation with $\boldsymbol{\sigma}^{+}$ polarization populates excitons and trions. Under the condition of electron doping, the resident electrons are polarized with spin down at the -K valley and therefore form more intervalley trions with resident electrons at the -K valley. **b,** Annihilation occurs between intervalley excitons and negative trions. The upper panel shows the process involving intervalley trions with resident electrons at the +K valley. The lower panel shows the process involving intervalley trions with resident electrons at the -K valley, which is the dominant mechanism. **c,** High-lying trions at the -K valley have a higher population than those at the +K valleys and show $\boldsymbol{\sigma}^{+}$ polarization.

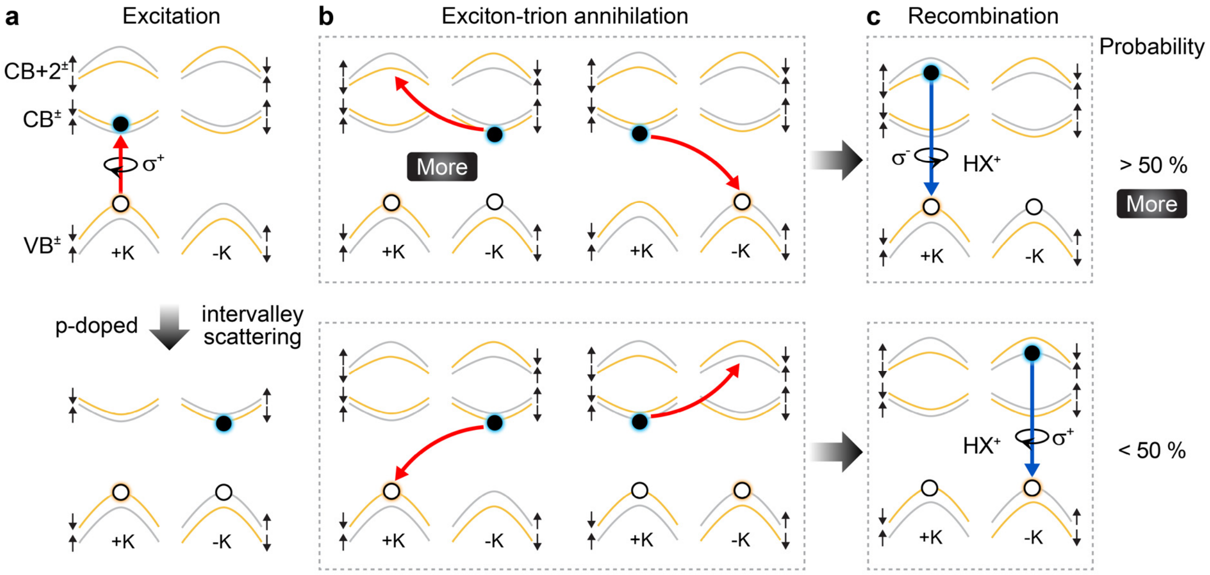

Supplementary Fig. 7 | Annihilation between an intervalley exciton and a trion for the formation of positive high-lying trions. a, Resonant excitation with $\boldsymbol{\sigma^+}$ polarization selectively populates trions at the +K valley. The top panel corresponds to the neutral regime. Through intervalley scattering, intervalley trions form with the resident hole at the -K valley. **b,** Annihilation occurs between intervalley excitons and positive trions. The upper panel shows the process involving intervalley trions with resident holes at the -K valley, which is the dominant one. The lower panel shows the process involving intervalley trions with resident holes at the +K valley. **c,** High-lying trions at the +K valley have a higher population than those at the -K valley and show $\boldsymbol{\sigma^-}$ polarization.

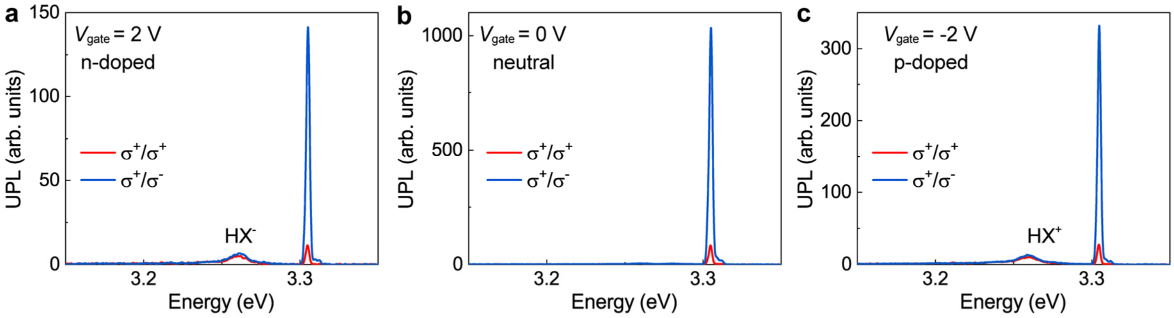

Supplementary Fig. 8 | Helicity of the SHG and the HX UPL from monolayer $MoSe_2$. Polarization-resolved SHG and UPL spectra from monolayer $MoSe_2$ at gate voltages of 2 V (a), 0 V (b), and -2 V (c). These spectra are identical to Fig. 3d-f but shown on a different scale.

Supplementary Note 3: Theoretical considerations of trions with negative-mass electrons

A. <u>Model of parabolic dispersion</u>

We consider an X^--trion formed of the hole with the effective mass m_h and two electrons with the effective masses m_1 and m_2. Let $m_1 > 0$ be the effective mass of the electron in the bottom conduction band and $m_2 < 0$ be the effective mass of the electron from the high-lying band. The effective Hamiltonian as a function of the relative electron-hole in-plane position-vectors $\boldsymbol{\rho}_1$ and $\boldsymbol{\rho}_2$ takes the form

$$\mathcal{H} = -\frac{\hbar^2}{2\mu_1}\Delta_1 - \frac{\hbar^2}{2\mu_2}\Delta_2 - \frac{\hbar^2}{m_h}\boldsymbol{\nabla}_1\boldsymbol{\nabla}_2 + V_{ee}(\boldsymbol{\rho}_1 - \boldsymbol{\rho}_2) + V_{eh}(\boldsymbol{\rho}_1) + V_{eh}(\boldsymbol{\rho}_2). \tag{1}$$

Here, subscripts 1 and 2 denote the differentiation between $\boldsymbol{\rho}_1$ and $\boldsymbol{\rho}_2$, respectively, and V_{ee} and V_{eh} are, respectively, the potential energies of the electron-electron repulsion and the electron-hole attraction. In Eq. (1) μ_1 and μ_2 are the reduced electron-hole masses defined as

$$\mu_1 = \frac{m_1 m_h}{m_1 + m_h}, \qquad \mu_2 = \frac{m_2 m_h}{m_2 + m_h}. \tag{2}$$

We recall that the total wavefunction of the trion can be represented as a product of the smooth envelope, which satisfies the Schrödinger equation with the Hamiltonian (1), and the three-particle Bloch function. The total wavefunction should be antisymmetric with respect to permutations of identical particles. Here, we focus on the states, where the Bloch function is antisymmetric and the envelope function is permutation-symmetric. These states usually dominate the optical response.

For symmetric trion states, where the two-electron envelope function $\psi_{tr}(\boldsymbol{\rho}_1, \boldsymbol{\rho}_2)$ is symmetric with respect to electron permutation[8-11], $\psi_{tr}(\boldsymbol{\rho}_1, \boldsymbol{\rho}_2) = \psi_{tr}(\boldsymbol{\rho}_2, \boldsymbol{\rho}_1)$, the quantum-mechanical average values of Δ_1 and Δ_2 are equal to each other:

$$\langle\Delta_1\rangle = \int d\boldsymbol{\rho}_1 d\boldsymbol{\rho}_2\, \psi_{tr}^*(\boldsymbol{\rho}_1, \boldsymbol{\rho}_2)\Delta_1\psi_{tr}(\boldsymbol{\rho}_1, \boldsymbol{\rho}_2) = \int d\boldsymbol{\rho}_1 d\boldsymbol{\rho}_2\, \psi_{tr}^*(\boldsymbol{\rho}_2, \boldsymbol{\rho}_1)\Delta_1\psi_{tr}(\boldsymbol{\rho}_2, \boldsymbol{\rho}_1) = \langle\Delta_2\rangle. \tag{3}$$

As a result, Eq. (1) is equivalent to the Hamiltonian

$$\mathcal{H} = -\frac{\hbar^2}{2\bar{\mu}}\left(\Delta_1 + \Delta_1 + \frac{2\sigma}{\sigma + 1}\boldsymbol{\nabla}_1\boldsymbol{\nabla}_2\right) + V_{ee}(\boldsymbol{\rho}_1 - \boldsymbol{\rho}_2) + V_{eh}(\boldsymbol{\rho}_1) + V_{eh}(\boldsymbol{\rho}_2). \tag{4}$$

with

$$\frac{1}{\bar{\mu}} = \frac{1}{2}\left(\frac{1}{\mu_1} + \frac{1}{\mu_2}\right), \qquad \sigma = \frac{\bar{\mu}}{m_h - \bar{\mu}} = \frac{2m_1m_2}{m_h(m_1 + m_2)}. \tag{5}$$

Thus, the problem of the high-lying trion maps to a problem of the trion with the effective reduced mass $\bar{\mu}$ and the electron-hole mass ratio σ.

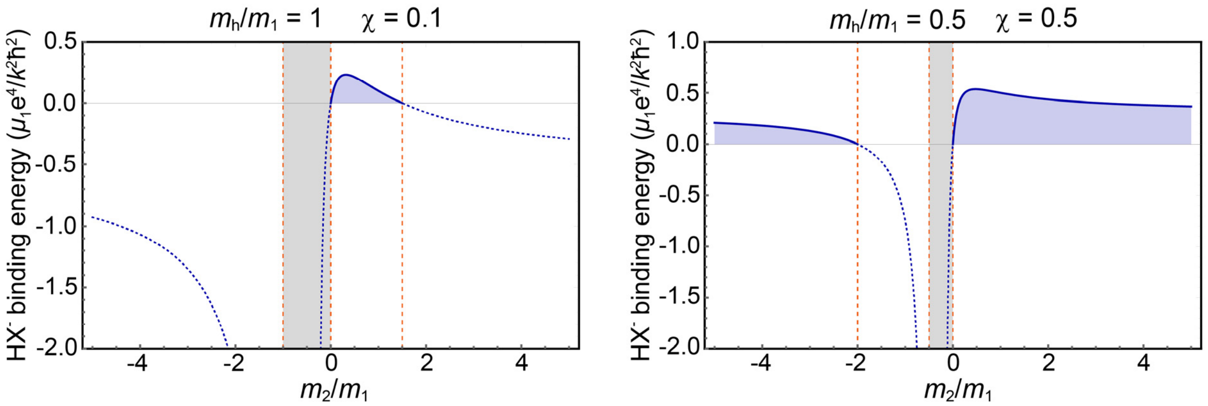

Supplementary Fig. 9 | High-lying trion binding energy calculated following Eq. (8) as a function of the m_2/m_1 mass ratio, plotted for two sets of parameters, where there is either no bound state ($m_2 < 0$, left) or a bound state is formed (right). Solid lines mark positive binding energy, where the trion is bound, i.e., where $E_{b,tr} > 0$ in Eq. (8), and dotted lines indicate negative binding energy. Gray shaded regions show the range of m_2 values for which μ_2 is negative and the corresponding high-lying exciton is unbound in the parabolic approximation. Orange lines mark the boundaries of the region where the trion is bound according to Eq. (9).

The trion binding energy is determined by the difference between the energy of the exciton formed by the hole and the high-lying negative-mass electron $\mathcal{E}_x = \min\langle -\frac{\hbar^2}{2\mu_2}\Delta_2 + V_{eh}(\boldsymbol{\rho}_2)\rangle$ and the total trion energy, found by the minimization of the Hamiltonian (4), $\mathcal{E}_{tr}^{tot} = \min\langle\mathcal{H}\rangle$, as

$$E_{\mathrm{b,HX^-}} = \mathcal{E}_x - \mathcal{E}_{tr}^{tot}. \tag{6}$$

The particular values of the trion binding energy can be determined, e.g., by variational calculations similar to those reported in Ref. 10. Here, we aim at establishing the bounds for the existence of the high-lying trion. To this end, we assume that $V_{ee}(\rho) = -V_{eh}(\rho) = e^2/\varkappa\rho$, with $\varkappa$ being the dielectric constant, i.e., we disregard the dielectric screening effects inherent to TMDC monolayers[12-15]. We have also checked that for the positive $\mu_1, \mu_2 > 0$ the account for the dielectric screening via the Rytova-Keldysh potential does not change the results qualitatively.

Calculations[8-10] show that the (total) trion energy can be written as

$$\mathcal{E}_{tr}^{tot} = -(1+\chi)\frac{2\bar{\mu}e^4}{\hbar^2\varkappa^2}, \tag{7}$$

where $\chi \equiv \chi(\sigma) \approx 0.1 \dots 0.5$, depending on σ, which describes the ratio of the trion to exciton binding energies in the case where the electron effective masses are the same. It follows from Eq. (6) that the trion binding energy is

$$E_{\mathrm{b,HX^-}} = -2\frac{\mu_2 e^4}{\hbar^2\varkappa^2} - \mathcal{E}_{tr}^{tot} = 2\frac{\mu_2 e^4}{\hbar^2\varkappa^2}\left[\frac{\bar{\mu}}{\mu_2}(1+\chi) - 1\right], \tag{8}$$

noting that that $\mathcal{E}_x = -2\mu_2 e^4/(\hbar^2\varkappa^2)$.

For the trion to be bound, the expression in the brackets should be positive, yielding the condition

$$\begin{cases} 0 < m_2 < m_* \equiv \dfrac{m_1 m_h(1+2\chi)}{m_h - 2\chi m_1}, \quad m_* > 0, \\ \quad 0 < m_2 \text{ or } m_2 < m_*, \quad m_* < 0. \end{cases} \tag{9}$$

It is worth noting that the reduced mass μ_2 should be positive, or else one has to go beyond the parabolic dispersion model to arrive at a bound high-lying exciton. In the case of the Rytova-Keldysh potential, the analysis can be performed in the same way, but the resulting numbers differ somewhat.

B. Effects of band non-parabolicity

Plots of the trion binding energy vs. m_2/m_1 are shown in Supplementary Fig. 9 for two sets of parameters. Note that for typical band-structure parameters the conditions (9) are not necessarily satisfied. However, the realistic dispersion in the CB+2 band is strongly non-parabolic and characterized both by quadratic and quartic terms[4,16,17].

To study the trion formation while accounting for non-parabolicity effects, neglecting anisotropy, we approximate the dispersion of the high-lying conduction band as

$$E_{2,k} = \frac{\hbar^2 k^2}{2m_2} + Bk^4, \quad m_2 < 0, \quad B > 0. \tag{10}$$

Accordingly, the dispersion for the high-lying exciton – the relative motion of electron and hole – takes the form

$$E_k = \frac{\hbar^2 k^2}{2\mu_2} + Bk^4, \quad B > 0. \tag{11}$$

The condition $B > 0$ implies the stability of the system, otherwise the high-lying exciton would acquire arbitrary negative energies. This condition is satisfied by the calculated band structure. We use the following improved trial functions for excitons

$$\psi_{HX}(\rho; a, b) \propto \begin{cases} \exp(-a\sqrt{b^2+\rho^2}), & \mu_2 > 0, \\ J_0(a\rho)\exp(-b\rho^2), & \mu_2 < 0. \end{cases} \tag{12}$$

We note that for $\rho \to 0$, the trial function should behave as ρ^2, otherwise the k^4 term diverges in the dispersion. This is why we used functions with corresponding asymptotes at $\rho \to 0$. Here, we

focus mainly on the experimentally relevant case with $\mu_2 > 0$; we therefore used a more accurate trial function with the Coulombic $\exp(-a\rho)$ asymptote at $\rho \to \infty$. The second function, where J_0 is the Bessel function, takes into account the specifics of the dispersion with the negative-mass situation, where the ground-state wavefunction oscillates as is seen from the scattering theory analysis, see subsection C below. With this wavefunction, the HX state is always bound, as follows from general theory.

The trion states are obtained by symmetrization of the exciton functions as

$$\psi_{HX^-} \propto \psi_{HX}(\rho_1; a_1, b_1)\psi_{HX}(\rho_2; a_2, b_2) + \psi_{HX}(\rho_2; a_1, b_1)\psi_{HX}(\rho_1; a_2, b_2). \quad (13)$$

The results of variational calculations are shown in Supplementary Fig. 10.

One can see that accounting for the quartic terms in the CB+2 dispersion results in the bound trion states forming over a wide range of parameters. At this stage, we abstain from a detailed comparison between experiment and theory and a concrete fitting of the binding energies. Such fitting would require accounting for the full dispersion of the CB+2 including its anisotropy and goes beyond the scope of the current work.

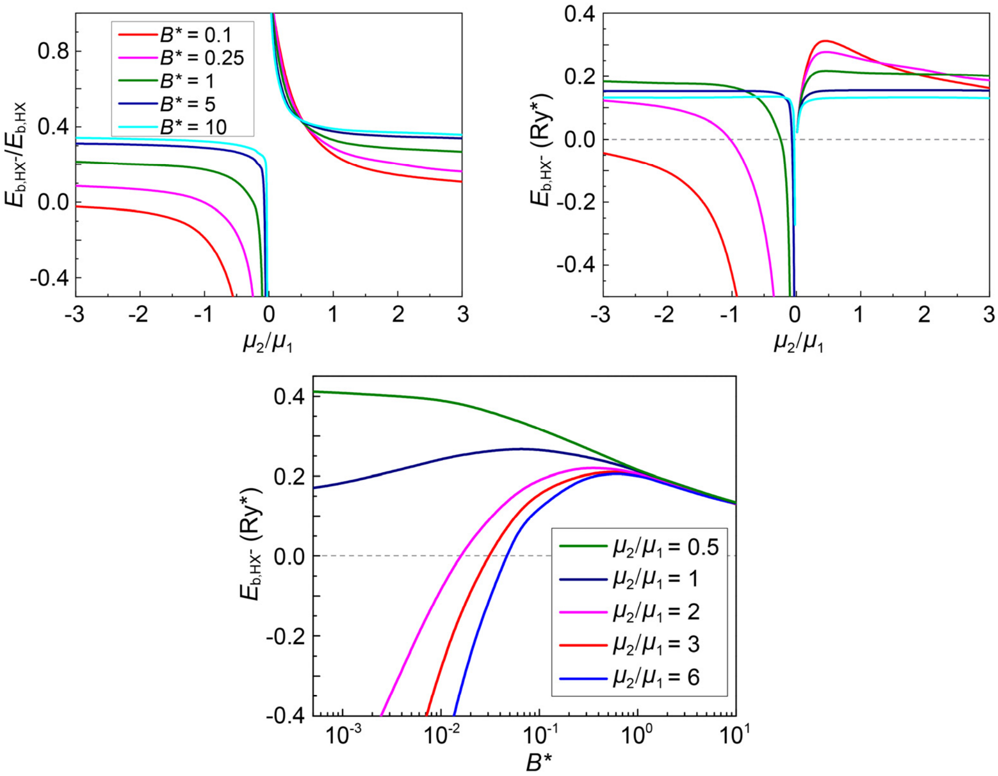

Supplementary Fig. 10 | Binding energies of the high-lying trion with improved trial functions. We set the system of units as $e = \mu_1 = \varkappa = 1$, where the energy is measured in $E = \mu_1 e^4/(\varkappa^2\hbar^2)$ and the distance is measured in $a = \varkappa\hbar^2/(\mu_1 e^2)$; $B^* = Be^4\mu_1^3/\varkappa^2\hbar^6$.

The results presented above were obtained using the variational method. To estimate its accuracy, we have performed additional calculations using the direct numerical diagonalization of the HX Hamiltonian on the basis of Gaussian wavefunctions. The results presented in Supplementary Fig. 11 demonstrate that both methods provide very close values of the exciton binding energy with the relative difference below ~1%. To calculate this figure, we also used Rytova-Keldysh potential and the results for the Coulomb potential converge in the same way. Note that the variational approach provides an upper bound for the total trion energy. Hence, since the trion binding energy is the difference between the total trion energy and the exciton energy, and the exciton energy is calculated to high accuracy, our variational calculation provides a lower bound for the trion binding energy – actual trion binding energies are expected to be higher as compared to the variational results in Supplementary Fig. 10. Our preliminary comparison with numerical calculations also shows that the error in the trion binding energy is small.

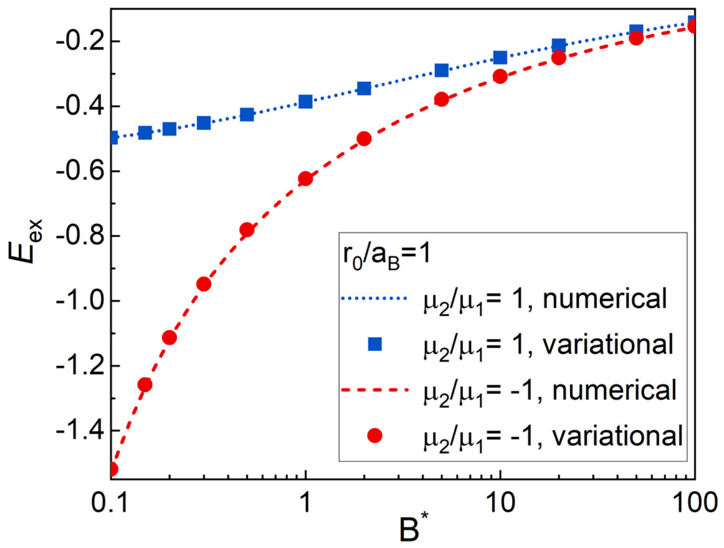

Supplementary Fig. 11 | Exciton energies in numerical diagonalization and variational approaches. Exciton energy (i.e. the binding energy with a negative sign) as a function of the quartic term in the dispersion $B^* = \mathrm{B}\frac{\mu_1^3 e^4}{\varkappa^2 \hbar^6}$, calculated using the variational approach (symbols) and using a numerical diagonalization of the Hamiltonian (lines) for two ratios of the reduced masses. The Rytova-Keldysh potential with the screening parameter $r_0 = a_B = 1.5\ nm$ was used in the calculation. The variational approach matches perfectly with the numerical calculation.

C. Scattering theory approach and high-lying Fermi polarons

In many cases, it is convenient to use an even more simplified model of the electron interaction with the exciton. Let us consider the high-lying exciton as a robust entity and take the dispersion for the relative motion between the high-lying exciton and the band-edge electron in the form similar to Eq. (11), where

$$E_k = \frac{\hbar^2 k^2}{2m} + Bk^4 = \frac{Ak^2}{2} + Bk^4, \quad B > 0, \tag{14}$$

with m being the exciton-electron reduced mass, and $A = \hbar^2/m$. Note that in this approach, m and A can be either positive or negative.

We assume that the exciton-electron interaction can be modelled by a sufficiently shortrange potential, with $V < 0$ being the interaction constant[1,18-21]. The bound state can be found from the pole of the scattering amplitude with the result

$$V \sum_{\boldsymbol{k}} \frac{1}{\mathcal{E} - E_k} = 1\,. \tag{15}$$

This equation at $A < 0$ can be transformed to

$$\arctan \frac{A}{\sqrt{-16B\mathcal{E} - A^2}} = \frac{\pi}{2} + \frac{\sqrt{-16B\mathcal{E} - A^2}}{2V}. \tag{16}$$

At $V \to 0$, from Eq. (16) we have $\mathcal{E} = -A^2/(16B) - (2\pi V)^2/(16B)$. The binding energy is the difference in energy between the minimum of the dispersion $-A^2/(16B)$ and $\mathcal{E}$, hence,

$$E_{\mathrm{b,HX^-}} = \frac{(\pi V)^2}{4B}, \;\; V \to 0. \tag{17}$$

At $V \to -\infty$, one has

$$E_{\mathrm{b,HX^-}} = \frac{(\pi V)^2}{16B} + \frac{AV}{4B}, \;\; V \to -\infty. \tag{18}$$

The corresponding dependence is shown in the left panel of Supplementary Fig. 12.

At $A > 0$, the asymptote at $V \to -\infty$ is given by Eq. (18), while the small-V asymptote follows from the relation

$$\ln\left(-\frac{4B\mathcal{E}}{A^4 - 4B\mathcal{E}}\right) = AV \;\Longrightarrow\; \mathcal{E} = \frac{A^2}{4B}\frac{1}{1 - \exp(-A/V)} \approx \frac{A^2}{4B} e^{A/V}. \tag{19}$$

Corresponding behavior of the binding energy for $A > 0$ is shown in the right panel of Supplementary Fig. 12.

Interestingly, if $A < 0$, the bound-state wavefunction is given by $\psi(r) \propto \sum_{\boldsymbol{k}} \frac{\exp(i\boldsymbol{kr})}{E_{\mathrm{b,HX^-}} + E_k}$, and it oscillates as a function of the distance r. This oscillation arises because of the quartic terms in the dispersion. This fact supports the selection of the trial function for the excitons in the form of Eq. (12), lower line, for $\mu_2 < 0$.

Such an approach allows us to apply the formalism of the exciton interaction with the Fermi sea of resident electrons. Following Refs. 18,19,21-23, one can describe the physics of the transfer of the oscillator strength between the high-lying exciton and trion (the Fermi polaron) as a function of the resident electron density. In particular, at small electron Fermi energies $E_F \ll E_{B,HX}$, the ratio of the high-lying trion to the exciton oscillator strength is given by the ratio[21]

$$\frac{f_{HT}}{f_{HX}} \sim \frac{E_F}{E_{\mathrm{b,HX^-}}}. \tag{20}$$

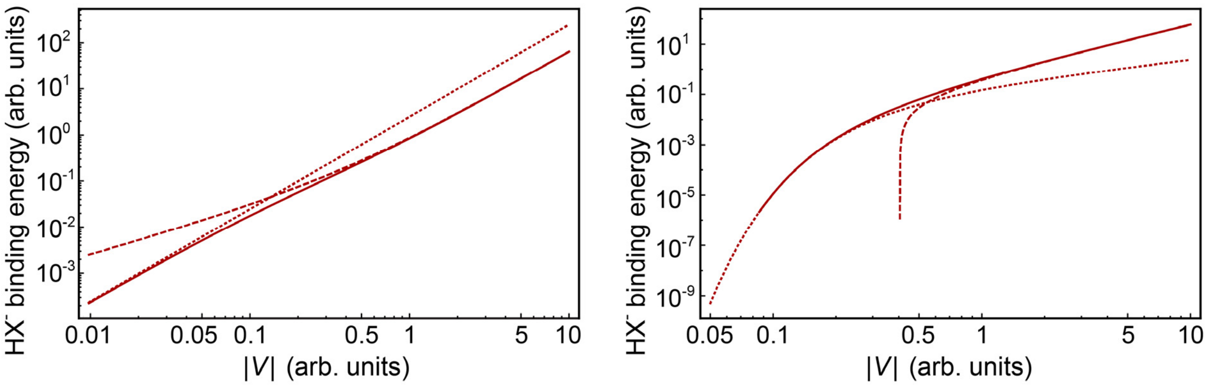

Supplementary Fig. 12 | High-lying trion binding energy found within the scattering-theory approach following Eq. (16). The left panel shows the case of $A < 0$. The asymptotic values for small (dotted line) and large (dashed line) absolute values of V are shown, following Eqs. (17) and (18). The right panel shows the case of $A > 0$ with the corresponding asymptotes calculated following Eqs. (18) and (19).

References

1 Wagner, K. *et al.* Autoionization and dressing of excited excitons by free carriers in monolayer WSe2. *Phys. Rev. Lett.* **125**, 267401 (2020).

2 Liu, E. *et al.* Exciton-polaron Rydberg states in monolayer MoSe2 and WSe2. *Nat. Commun.* **12**, 6131 (2021).

3 Mak, K. F. *et al.* Tightly bound trions in monolayer MoS2. *Nat. Mater.* **12**, 207 (2012).

4 Lin, K.-Q. *et al.* Narrow-band high-lying excitons with negative-mass electrons in monolayer WSe2. *Nat. Commun.* **12**, 5500 (2021).

5 Goryca, M. *et al.* Revealing exciton masses and dielectric properties of monolayer semiconductors with high magnetic fields. *Nat. Commun.* **10**, 4172 (2019).

6 Erkensten, D. *et al.* Dark exciton-exciton annihilation in monolayer WSe2. *Phys. Rev. B* **104**, L241406 (2021).

7 Robert, C. *et al.* Spin/valley pumping of resident electrons in WSe2 and WS2 monolayers. *Nat. Commun.* **12**, 5455 (2021).

8 Sergeev, R. A. & Suris, R. A. Ground-state energy of X− and X+ trions in a two-dimensional quantum well at an arbitrary mass ratio. *Phys. Solid State* **43**, 746-751 (2001).

9 Berkelbach, T. C., Hybertsen, M. S. & Reichman, D. R. Theory of neutral and charged excitons in monolayer transition metal dichalcogenides. *Phys. Rev. B* **88**, 045318 (2013).

10 Courtade, E. *et al.* Charged excitons in monolayer WSe2: experiment and theory. *Phys. Rev. B* **96**, 085302 (2017).

11 Semina, M. A. Excitons and trions in bilayer van der Waals heterostructures. *Phys. Solid State* **61**, 2218-2223 (2019).

12 Rytova, N. S. The screened potential of a point charge in a thin film. *Mosc. Univ. Phys. Bull.* **3**, 18 (1967).

13 Keldysh, L. V. Coulomb interaction in thin semiconductor and semimetal films. *Sov. J. Exp. Ther. Phys. Lett.* **29**, 658 (1979).

14 Cudazzo, P., Tokatly, I. V. & Rubio, A. Dielectric screening in two-dimensional insulators: Implications for excitonic and impurity states in graphane. *Phys. Rev. B* **84**, 085406 (2011).

15 Chernikov, A. *et al.* Exciton binding energy and nonhydrogenic Rydberg series in monolayer WS2. *Phys. Rev. Lett.* **113**, 076802 (2014).

16 Kormányos, A. *et al.* k·p theory for two-dimensional transition metal dichalcogenide semiconductors. *2D Mater.* **2**, 022001 (2015).

17 Durnev, M. V. & Glazov, M. M. Excitons and trions in two-dimensional semiconductors based on transition metal dichalcogenides. *Phys.-Usp.* **61**, 825-845 (2018).

18 Suris, R. A. in *Optical Properties of 2D Systems with Interacting Electrons* (eds Wolfgang J. Ossau & Robert Suris) 111-124 (Springer, 2003).

19 Sidler, M. *et al.* Fermi polaron-polaritons in charge-tunable atomically thin semiconductors. *Nat. Phys.* **13**, 255-261 (2017).

20 Fey, C., Schmelcher, P., Imamoglu, A. & Schmidt, R. Theory of exciton-electron scattering in atomically thin semiconductors. *Phys. Rev. B* **101**, 195417 (2020).

21 Glazov, M. M. Optical properties of charged excitons in two-dimensional semiconductors. *J. Chem. Phys.* **153**, 034703 (2020).

22 Schmidt, R., Enss, T., Pietilä, V. & Demler, E. Fermi polarons in two dimensions. *Phys. Rev. A* **85**, 021602 (2012).
23 Efimkin, D. K. & MacDonald, A. H. Many-body theory of trion absorption features in two-dimensional semiconductors. *Phys. Rev. B* **95**, 035417 (2017).
